# Supplementary material for: Frequencies and functions of vocalizations and gestures in the second year of life
Source: PLoS One. 2025 Jan 9;20(1):e0308760. doi: 10.1371/journal.pone.0308760 (PMC11717318; doi:10.1371/journal.pone.0308760)
Supplement: S1 Appendix — (DOCX) [file pone.0308760.s001.docx]

Appendix Table: **GEE Models**

**Hypothesis 1a: Nonsocial Gestures v. Nonsocial Vocalizations across Age**

Age as factors model: Age 3 is the baseline

Nonsocial Gesture vs Nonsocial Vocalization : Vocalization is the baseline

| Coefficient | Estimate (B) | std err | p-value |
| --- | --- | --- | --- |
| Intercept | .791 | .2432 | .001 |
| Age 13 mo. | .418 | .2523 | .097 |
| Age 16 mo. | -.120 | .1900 | .528 |
| Modality | -.688 | .2145 | .001 |
| Age13*Modality | -.368 | .2268 | .104 |
| Age16*Modality | .238 | .1980 | .230 |

**Hypothesis 1b: Universal Gestures v. Universal Vocalizations across Age**

Age as factors model: Age 3 is the baseline

Universal Gesture vs Universal Vocalization : Vocalization is the baseline

| Coefficient | Estimate (B) | std err | p-value |
| --- | --- | --- | --- |
| Intercept | .809 | .1317 | <.001 |
| Age 13 mo. | 1.152 | .4281 | .007 |
| Age 16 mo. | .431 | .3471 | .215 |
| Modality | 1.070 | .4462 | .016 |
| Age13*Modality | -1.858 | .7429 | .012 |
| Age16*Modality | -.598 | .5313 | .260 |

**Hypothesis 1c: Conventional Gesture v. Conventional Vocalization across Age**

Age as factors model: Age 3 is the baseline

Conventional Gesture vs Conventional Vocalization : Vocalization is the baseline

| Coefficient | Estimate (B) | std err | p-value |
| --- | --- | --- | --- |
| Intercept | 3.338 | .5909 | <.001 |
| Age 13 mo. | -2.091 | .6294 | <.001 |
| Age 16 mo. | 1.245 | .6488 | .055 |
| Modality | -3.173 | .5820 | <.001 |
| Age13*Modality | 2.197 | .6281 | <.001 |
| Age16*Modality | 1.337 | .6628 | .044 |

**Analysis 2: Proportion of Directed Gestures v. Proportion of Directed Vocalizations across Age**

Age as factors model: Age 3 is the baseline

Gesture vs. Vocalization: Vocalization is the baseline

| Coefficient | Estimate (B) | std err | p-value |
| --- | --- | --- | --- |
| Intercept | .853 | .0311 | <.001 |
| Age 13 mo. | -.088 | .0441 | .047 |
| Age 16 mo. | -.042 | .0419 | .318 |
| Modality | -.048 | .0334 | .153 |
| Age13*Modality | -.107 | .0795 | .179 |
| Age16*Modality | -.016 | .0546 | .767 |
