# Supplementary material for: Frequencies and functions of vocalizations and gestures in the second year of life
Source: PLoS One. 2025 Jan 9;20(1):e0308760. doi: 10.1371/journal.pone.0308760 (PMC11717318; doi:10.1371/journal.pone.0308760)
Supplement: Attachment — Submitted filename: PLoS One Sep 2023.pdf [file pone.0308760.s002.pdf]

The manuscript consists of a timely study that challenges gesture-first theories of language. The paper begins with a review of the literature, suggesting that gesture-first theories are in the ascendency. The authors then argue that on the contrary, it is more plausible that humans had selection pressure to use vocalisations more (compared at least to other primates). The main hypothesis of the paper is that if earliest language was gestural – we should expect extensive gestural use early in development, whereas if vocal – we should expect extensive vocal use in development. Comparisons between gestures and vocalisation (on any metric) are difficult, so I appreciate the authors descriptive framework (universal, non-social and conventional vocalisations/gestures). The sample size is quite small, but the observation time is densely sampled (60 mins total per participant across three sessions), so this is still very informative. The results suggest that (a) non-social vocalisations are far more frequent than non-social gestures (which does not change across the second year of life), (b) neither universal vocalisations and gestures were more frequent across the second year of life, however the tendency was that vocalisations decreased across the year and gestures increased, (c) conventional vocalisations were more frequent and increased across the second year of life, while conventional gestures were infrequent and did not increase/decrease over time, and finally (d) all types of gestures were more likely to be directed to caregivers (as indexed by gaze) than vocalisations across the second year.

The paper is well written and important. I agree with the authors that clearly the claim that early gesture use exceeds vocalisation use in human infancy is unfounded in light of this (and other data). I especially enjoyed the discussion of action gestures – and share the authors concern that "true gestures" are often confounded with actions (that are not gestures) simply because they could one day be ritualised into gestures, but that does not mean that they are true gestures when first used by infants! The same concern might also be applied to chimpanzee gesture coding schemes (which often include manual actions, e.g., splashing, as gestures…). I do however have a number of concerns about the manuscript in its current form, detailed below. I think that all the issues I raise are surmountable and very much look forward to reading a revised manuscript.

Introduction and literature review

There is a missing step in the introduction. I agree that there are proponents of gesture-first theories (and there is a sense at which many in the field simply uncritically appear to accept it as a premise), but there also vocal detractors (excuse the pun). It would be of benefit to the introduction to include an extra section detailing the body of opinion and evidence already amassed that disputes gesture-first theories (in the Vocalization and Gesture in Communicative Evolution section) before launching into the evolutionary argument (Natural Selection of Communicative Signals). For example, there was a review paper suggesting that great ape data simply doesn't support a gesture-first *or* vocal-first origin story (Frohlich et al., 2019 *https://doi.org/10.1111/brv.12535*). On the developmental side of things, there are other papers that provide evidence that would already count against gesture-first theories (e.g., McGillion et al., *https//doi.org/10.1111/cdev.12671* ; Donnellan et al., 2020 *https://doi.org/10.1111/desc.12843*; McCune et al., 2020 *https://doi.org/10.1177/014272372096682*; Werwach et al., 2021 *https://doi.org/10.1016/j.infbeh.2021.10158*). Inclusion of a review of the literature that already supports the authors main contention would strengthen the manuscript.

Universal vocalisations

I am unconvinced by the justification in the current manuscript that crying, whimpering and laughter should be included as universal vocalisations. Universal gestures are defined with respect to communicative intent, however it's not clear that laughter, crying and whimpering necessarily are produced with communicative intent (though it's possible – and of course whether deictic gestures are always produced with communicative intent is debatable too [examples of children pointing for themselves, see Delgado, Gómez, & Sarriá, 2011: https://doi.org/10.1016/j.jecp.2011.04.010]). I am much more convinced that non-word conversational protophones should be included as universal vocalisations – both these and deictic gestures promote engagement with caregivers (and more richly, could be intended for this purpose on the part of the infant – though this is speculative [but often a speculation accorded to gestures only in the literature…]). I don't quite see the parallels between deictic gestures with affective vocalisations – I do take the authors point that they have a modality-specific

function (expressing affect -which gestures can't do without a lexicon), but that doesn't seem a good enough reason to group them together, especially because they are also grouped with vocalisations that might not have a modality-specific function (e.g., the conversational protophones – whose function [promoting engagement] could also be achieved gesturally [e.g., through showing: holding up objects to caregivers face, or even pointing at caregivers]). I think as it stands, universal vocalisations should consist *either* of crying, whimpering and laughter (to maintain the modality-specific function element) *or* conversational protophones (to capture the engagement element). As is stands, the justification is clearer to me that it should just be the protophones.

Measures

Sessions are approximately 20 mins (not exactly 20 mins). This makes it hard to interpret individual differences across participants. How close to 20 minutes are the sessions? If within a few seconds, this is fine. However, if discrepancies are significant, behaviours should be standardized as rates per minute (or rate per 20 mins if preferred) instead of as a raw frequency. The analysis should then be re-run with these standardized measures.

Reliability Coding

The first paragraph of Coder Agreement and Outcomes section is unnecessary – this is simply reporting reliability statistics from a previous study – not the current study – and it's not clear why this is relevant. I suggest simply omitting this.

The final paragraph concerning categorisation of vocalization and gesture into the three functional categories requires more quantitative reliability metrics. I agree (by eyeballing the plot) that this probably shows pretty decent agreement, however, given the way in which the data was coded, it should be possible to calculate kappa reliability (if I have understood the coding procedure correctly). I.e., for the instances in which both coders agreed there was a vocalisation or a gesture – did they agree on the function (i.e., did they both select an illocution code that would be categorized as the same function?).

Overall though, it should be noted that reliability correlations currently reported are solid (and my impression is that the kappas will equally demonstrate this) – I have no doubt that this coding has been conducted meticulously, but there do need to be missing metrics included to confirm this.

Analysis

GEE does seem an appropriate method for the data, and likewise Mann-Whitney tests are appropriate in my understanding. The GEE models obtained need to be included in the paper in full (if even simply in tables in an Appendix).

Data visualisation

Given that the authors rely heavily on the visualisation of the data to do much of the work of inference (and correctly so I think!), the visualisations need work. I have a few suggestions:

Distributions of data: Ideally the distribution of the datapoints themselves needs to be included (not just 95% confidence intervals and a mean). Numerous ways exist to do this, for example violin plots, half violin plots, and/or plotting the individual data points. E.g., in figures 3, 4 and 5, individual points (representing a participant) could be plotted in red at each time point for vocalisations and in blue for gestures, with summary statistics (mean/CIs) overlaid, perhaps in tandem with violin plots or similar.

Model predictions. Equally, if GEE was implemented using an R package, it may be possible to plot model predictions (perhaps overlaid) on the figures automatically – which would be very informative. Of course it is possible to do manually, but this does require expertise. This is becoming much more of a standard approach to visualising data (i.e., raw scores – as you have here, overlaid with model estimates). This would really support the authors inferences from the data and I would strongly encourage that they

attempt this. This would also remove the need for caveats to be included in the figure captions about what the model shows compared to what the figure seems to show (i.e., the confidence intervals relating to the the raw data not the SE/confidence intervals of the model predictions).

Figures 2 and 5. For hypothesis 2 a pie chart is not informative – as the data comes from individual participants. Please plot the data so that the range of proportions of gestures/vocalisations combined with eye gaze across participants is clear (i.e., in the same way as figures relating to hypothesis 1 would be fine, y axis being proportion). As currently plotted, the discrepancy between the data and the model results (especially with regard to 20m timepoints) is hard to interpret (i.e., why there is a main effect of modality, not an interaction with age). Likewise for figure 2, though there are error bars, perhaps consider plotting the individual data points with summary statistics overlaid as suggested above (i.e., not as a bar chart), so the distribution of individual participant data can be observed.

Data availability

Currently the data for the study is not available. I agree fully that videos cannot be shared. It is perfectly possible however to have the data worksheet mentioned in the availability statement already uploaded into the OSF for example (it can be a private submission), with an anonymized link provided only for the purpose of review.

Minor comments

Fig 3. - Typo on y axis label ("Frequancy"). Also figure 3 error bars do not look correct and it's a screenshot containing "plot area" in a box over the middle. I have suggested more substantial changes to figures above.

Typo – in the Results paragraph headed "Hypothesis 1a", the text refers to Figure 5 – this should be figure 3?

Please include in the limitations section that this is still a relatively small sample size (in terms of unique participants).

Please include line numbers in the next draft to facilitate navigating the text and providing feedback.

When discussing possibility of vocalization to serve function of communication very early in development (e.g., page 2) consider citing Papaeliou et al., 2002 (10.1044/1092-4388(2002/024)) and/or Esteve-Gibert & Prieto 2012 (https://doi.org/10.1017/S0305000912000359) as these seem relevant too.

Note, I could not see tables in my copy of the manuscript. Whether this is an editorial mistake or submission mistake, I'm unsure (I contacted the journal to ask, but didn't get a response before submitting my review to the deadline). I look forward to reviewing the content of the tables in the next round of review.
