# Supplementary material for: Frequencies and functions of vocalizations and gestures in the second year of life
Source: PLoS One. 2025 Jan 9;20(1):e0308760. doi: 10.1371/journal.pone.0308760 (PMC11717318; doi:10.1371/journal.pone.0308760)
Supplement: Attachment — Submitted filename: PLOS VOCALIZATIONS AND GESTURES.doc [file pone.0308760.s003.doc]

Journal: 	OPEN LINGUISITCS JOURNAL

Article No:

Author(s):	NA

PEER REVIEW- 15/10/2023

Dear Editor,

**GENERAL**

I would like to thank you for giving me the opportunity to review this interesting and well-written piece of work. As per your request, here is the review of PONE-D-23-20708 entitled Frequencies and functions of vocalizations and gestures in the second year of life”. My recommendation is “Acceptance with minor changes”. My comments are mostly related to methodological and stylistic issues. The paper focuses on the critical theme related to the importance of gestures vis a vis vocalization as early indications of the origins of language. It follows a previously conducted study, with the current paper adding and supporting evidence of their theoretical EVO-DEVO framework. The theoretical framework of observation highlights the fact that more than ¾ of gestures across these second-year data were deictics (e.g., pointing and reaching), acts that while significant in supporting the establishment of referential vocabulary in both spoken and signed languages, are not signs, but have single universal deictic functions in the here and now. In contrast, words and signs are functionally flexible, making possible reference to abstractions that are not bound to any “particular illocutionary force nor to the here and now”. This topic is long overdue given the scarcity of data available from such topics related to the origins of languages and dialects.

I remain at your disposal

Yours sincerely

Professor Kakia Petinou, PhD -SLP

Correspondence details:

Work address: Cyprus University of Technology, Vragadinou 15 Zt3

Limassol 3036, Cyprus

Tel: +35799409449

Email:kakia.petinou@cut.ac.cy

**INTRODUCTION**

The literature review is relevant. The paper is clearly written with bibliographic references on par with latest published research. On page 14 the authors provide a clear description of the different types of gestures. I was glad to this this section because it provided a clear pathway to the information following the introduction.

I suggest that the authors provide the term typically developing (experimental group) earlier in the paper.

**METHODOLOGY**

I am not clear on how toddlers were examined to exclude developmental/language challenges. Dis children have any episodes of otitis media? Where they excluded? I mention this because under such circumstances even children and toddlers with neurotypical development use more communicative gestures to compensate for reduced vocal output and compromised hearing sensitivity.

Is there a rater-to-rater reliability score?

**RESULTS**

No comments. This is a qualitative examination and data are clearly presented in a via descriptive non-param stats.

**DISCUSSION**

The authors describe the important performance and provide adequate explanation of the findings. The writing is clear and the manuscript is well-organized.

The authors provide adequate description of findings which are combined with plausible interpretations.

The novelty of the study rests on the fact that the findings contribute and enrich the database related to vocalizations versus gestures.

-------------------------
