# Supplementary material for: Frequencies and functions of vocalizations and gestures in the second year of life
Source: PLoS One. 2025 Jan 9;20(1):e0308760. doi: 10.1371/journal.pone.0308760 (PMC11717318; doi:10.1371/journal.pone.0308760)
Supplement: Attachment — Submitted filename: response to reviewers.docx [file pone.0308760.s004.docx]

**See our responses below in boldface.**

Dear Dr. Burkhardt-Reed,

Thank you for submitting your manuscript to PLOS ONE. After careful consideration, we feel that it has merit but does not fully meet PLOS ONE’s publication criteria as it currently stands. Therefore, we invite you to submit a revised version of the manuscript that addresses the points raised during the review process.

Upon careful review of the text and the reviewers' comments, it is evident that it is a valuable addition to the field of research it pertains to and is potentially worthy of publication.You should focus on addressing the comments made by Rev. 1 regarding the introduction and taking into account the methodological issues raised by both reviewers. Additionally, the way the data is visualized needs to be improved. You are advised to refer to the Rev1 comments for more detailed contributions.

Please submit your revised manuscript by Dec 01 2023 11:59PM. If you will need more time than this to complete your revisions, please reply to this message or contact the journal office at [plosone@plos.org](mailto:plosone@plos.org). When you're ready to submit your revision, log on to <https://www.editorialmanager.com/pone/> and select the 'Submissions Needing Revision' folder to locate your manuscript file.

Please include the following items when submitting your revised manuscript:

- A rebuttal letter that responds to each point raised by the academic editor and reviewer(s). You should upload this letter as a separate file labeled 'Response to Reviewers'.
- A marked-up copy of your manuscript that highlights changes made to the original version. You should upload this as a separate file labeled 'Revised Manuscript with Track Changes'.
- An unmarked version of your revised paper without tracked changes. You should upload this as a separate file labeled 'Manuscript'.

If you would like to make changes to your financial disclosure, please include your updated statement in your cover letter. Guidelines for resubmitting your figure files are available below the reviewer comments at the end of this letter.

We look forward to receiving your revised manuscript.

Kind regards,

Elena Theodorou
Academic Editor
PLOS ONE

Journal Requirements:

When submitting your revision, we need you to address these additional requirements.

1. Please ensure that your manuscript meets PLOS ONE's style requirements, including those for file naming. The PLOS ONE style templates can be found at

<https://journals.plos.org/plosone/s/file?id=wjVg/PLOSOne_formatting_sample_main_body.pdf> and 
<https://journals.plos.org/plosone/s/file?id=ba62/PLOSOne_formatting_sample_title_authors_affiliations.pdf>

**The manuscript has been updated to reflect PLOS ONE’s style requirements in the main body and title page.**

2. Note from Emily Chenette, Editor in Chief of PLOS ONE, and Iain Hrynaszkiewicz, Director of Open Research Solutions at PLOS:

Did you know that depositing data in a repository is associated with up to a 25% citation advantage (<https://doi.org/10.1371/journal.pone.0230416>)? If you’ve not already done so, consider depositing your raw data in a repository to ensure your work is read, appreciated and cited by the largest possible audience. You’ll also earn an Accessible Data icon on your published paper if you deposit your data in any participating repository (<https://plos.org/open-science/open-data/#accessible-data>).

**We have chosen Mendeley,** [**https://data.mendeley.com/preview/fk6gfr65p9?a=64fb05c6-fc70-4227-9d28-6bfb19593a96**](https://data.mendeley.com/preview/fk6gfr65p9?a=64fb05c6-fc70-4227-9d28-6bfb19593a96)

3. Please provide additional details regarding participant consent. In the ethics statement in the Methods and online submission information, please ensure that you have specified what type you obtained (for instance, written or verbal, and if verbal, how it was documented and witnessed). If your study included minors, state whether you obtained consent from parents or guardians. If the need for consent was waived by the ethics committee, please include this information.

**Done in the revised ms.**

Once you have amended this/these statement(s) in the Methods section of the manuscript, please add the same text to the “Ethics Statement” field of the submission form (via “Edit Submission”).

**The ethics statement has been updated upon re-submission.**

For additional information about PLOS ONE ethical requirements for human subjects research, please refer to <http://journals.plos.org/plosone/s/submission-guidelines#loc-human-subjects-research>.

4. In your Data Availability statement, you have not specified where the minimal data set underlying the results described in your manuscript can be found. PLOS defines a study's minimal data set as the underlying data used to reach the conclusions drawn in the manuscript and any additional data required to replicate the reported study findings in their entirety. All PLOS journals require that the minimal data set be made fully available. For more information about our data policy, please see <http://journals.plos.org/plosone/s/data-availability>.

Upon re-submitting your revised manuscript, please upload your study’s minimal underlying data set as either Supporting Information files or to a stable, public repository and include the relevant URLs, DOIs, or accession numbers within your revised cover letter. For a list of acceptable repositories, please see <http://journals.plos.org/plosone/s/data-availability#loc-recommended-repositories>. Any potentially identifying patient information must be fully anonymized.

**Mendeley**

Important: If there are ethical or legal restrictions to sharing your data publicly, please explain these restrictions in detail. Please see our guidelines for more information on what we consider unacceptable restrictions to publicly sharing data: <http://journals.plos.org/plosone/s/data-availability#loc-unacceptable-data-access-restrictions>. Note that it is not acceptable for the authors to be the sole named individuals responsible for ensuring data access.

**Recordings cannot be shared because of confidentiality constraints for the human subjects. But the data will be deposited in Mendeley for public sharing.**

We will update your Data Availability statement to reflect the information you provide in your cover letter."""

5. We note that you have stated that you will provide repository information for your data at acceptance. Should your manuscript be accepted for publication, we will hold it until you provide the relevant accession numbers or DOIs necessary to access your data. If you wish to make changes to your Data Availability statement, please describe these changes in your cover letter and we will update your Data Availability statement to reflect the information you provide.

**Mendeley, https://data.mendeley.com/preview/fk6gfr65p9?a=64fb05c6-fc70-4227-9d28-6bfb19593a96**

6. Please upload a copy of Figure 7, to which you refer in your text on page 30 (in PDF format). If the figure is no longer to be included as part of the submission please remove all reference to it within the text.

**This Figure was submitted, but somehow did not reach one or both reviewers. We will be even more careful to ensure its presence in the revision in a separate PDF. It is now labeled correctly as Figure 5.**

7. Please include a copy of Tables 1 to 3 which you refer to in your text on pages 18 and 21 (in PDF format).

**Again these were originally submitted but somehow never reached the reviewers. All tables and figures have been provided in a separate PDF.**

Reviewers' comments:

Reviewer 1

The manuscript consists of a timely study that challenges gesture-first theories of language. The paper begins with a review of the literature, suggesting that gesture-first theories are in the ascendency. The authors then argue that on the contrary, it is more plausible that humans had selection pressure to use vocalisations more (compared at least to other primates). The main hypothesis of the paper is that if earliest language was gestural – we should expect extensive gestural use early in development, whereas if vocal – we should expect extensive vocal use in development. Comparisons between gestures and vocalisation (on any metric) are difficult, so I appreciate the authors descriptive framework (universal, non-social and conventional vocalisations/gestures). The sample size is quite small, but the observation time is densely sampled (60 mins total per participant across three sessions), so this is still very informative. The results suggest that (a) non-social vocalisations are far more frequent than non-social gestures (which does not change across the second year of life), (b) neither universal vocalisations and gestures were more frequent across the second year of life, however the tendency was that vocalisations decreased across the year and gestures increased, (c) conventional vocalisations were more frequent and increased across the second year of life, while conventional gestures were infrequent and did not increase/decrease over time, and finally (d) all types of gestures were more likely to be directed to caregivers (as indexed by gaze) than vocalisations across the second year. The paper is well written and important. I agree with the authors that clearly the claim that early gesture use exceeds vocalisation use in human infancy is unfounded in light of this (and other data). I especially enjoyed the discussion of action gestures – and share the authors concern that “true gestures” are often confounded with actions (that are not gestures) simply because they could one day be ritualised into gestures, but that does not mean that they are true gestures when first used by infants! The same concern might also be applied to chimpanzee gesture coding schemes (which often include manual actions, e.g., splashing, as gestures…). I do however have a number of concerns about the manuscript in its current form, detailed below. I think that all the issues I raise are surmountable and very much look forward to reading a revised manuscript. Introduction and literature review There is a missing step in the introduction. I agree that there are proponents of gesture-first theories (and there is a sense at which many in the field simply uncritically appear to accept it as a premise), but there also vocal detractors (excuse the pun). It would be of benefit to the introduction to include an extra section detailing the body of opinion and evidence already amassed that disputes gesture-first theories (in the Vocalization and Gesture in Communicative Evolution section) before launching into the evolutionary argument (Natural Selection of Communicative Signals). For example, there was a review paper suggesting that great ape data simply doesn’t support a gesture-first or vocal-first origin story (Frohlich et al., 2019 <https://doi.org/10.1111/brv.12535>). } On the developmental side of things, there are other papers that provide evidence that would already count against gesture-first theories (e.g., McGillion et al., https//doi.org/10.1111/cdev.12671; Donnellan et al., 2020 <https://doi.org/10.1111/desc.12843>; McCune et al., 2020 <https://doi.org/10.1177/014272372096682>; Werwach et al., 2021 <https://doi.org/10.1016/j.infbeh.2021.10158>). Inclusion of a review of the literature that already supports the authors main contention would strengthen the manuscript.

Universal vocalisations I am unconvinced by the justification in the current manuscript that crying, whimpering and laughter should be included as universal vocalisations. Universal gestures are defined with respect to communicative intent, however it’s not clear that laughter, crying and whimpering necessarily are produced with communicative intent (though it’s possible – and of course whether deictic gestures are always produced with communicative intent is debatable too [examples of children pointing for themselves, see Delgado, Gómez, & Sarriá, 2011: <https://doi.org/10.1016/j.jecp.2011.04.010>]).

I am much more convinced that non-word conversational protophones should be included as universal vocalisations – both these and deictic gestures promote engagement with caregivers (and more richly, could be intended for this purpose on the part of the infant – though this is speculative [but often a speculation accorded to gestures only in the literature…]). I don’t quite see the parallels between deictic gestures with affective vocalisations – I do take the authors point that they have a modality-specific function (expressing affect -which gestures can’t do without a lexicon), but that doesn’t seem a good enough reason to group them together, especially because they are also grouped with vocalisations that might not have a modality-specific function (e.g., the conversational protophones – whose function [promoting engagement] could also be achieved gesturally [e.g., through showing: holding up objects to caregivers face, or even pointing at caregivers]). I think as it stands, universal vocalisations should consist either of crying, whimpering and laughter (to maintain the modality-specific function element) or conversational protophones (to capture the engagement element). As is stands, the justification is clearer to me that it should just be the protophones.

**We greatly appreciate these comments about the categorization of universal gestures and vocalizations. We have reconsidered our categorization system in response to R1’s thoughts and have modified the text of the paper accordingly.**

**Deictic gestures are not necessarily “parallel” with affective vocalizations (those that transmit positive or negative affective valences), but both deictic gestures and affective vocalizations universal, and that is our point. Deictic gestures have been routinely treated as important in communication, but affective vocalizations have not been treated as a such even though they constitute a natural counterweight to deictic gestures in assessing the roots of linguistic communication.**

**Still we understand R1’s concern that cry and laughter might not be viewed as intentional in the same way as any of the other communicative actions considered in our paper. We take up this issue below offering perspective on why cry and laughter are often, if not usually, intentional as communications at least in the second year.**

**But first, we were struck by something we noticed during the new scrutiny of our findings (we’re surprised we hadn’t noticed it before), namely that the amount of crying, whimpering and laughter that occurred in the sample was small, only 13% of the total vocalizations that entered into the universal counts we provided, and the great majority of that proportion (69%) was laughter. The argument we presented concerning the theoretical importance of including crying, whimpering and laughter in the universal counts is thus largely moot empirically. We can, although it would take some recalculating, provide an analysis to put in an Appendix that excludes all the cries, whimpers, and laughs from any of the categories if the editor and R1 think it would be important. We prefer, however, to maintain the argument for inclusion of crying, whimpering and laughter as in the original analysis.**

**There remains a different quandary though. R1 seemed satisfied that conversational protophones should be included in the universals, but the coding included quite a number of protophones that were affectively negative (whining complaints that we interpreted as attempts by the infant to gain attention or modify the caregiver’s actions in some way) or affectively positive (interactively exultative sounds accompanied by smiling that we interpreted as encouragements by the infants for the caregivers to interact). These affectively positive or negative protophones *were coded as universals*. Other protophones (but less than half the protophones we included in the universal counts) were responsive to parental vocal elicitation without facial signs of positive or negative affect, and so could be viewed as strictly conversational.**

**As for intentionality, which seemed to play a significant role in R1’s concern about the categorization of crying, whimpering and laughter as universals, there is considerable evidence (and it has been on the table for decades) that laughter in infants and young children is overwhelmingly socially interactive** **[1-3]. We have found this to be true in our own observations, that for the last author have included 7 longitudinal studies over a period of 5 decades. Also a renewed look at our present data showed that laughter accounted for the great majority of the crying, whimpering and laughter (69% of it). We agree that crying and whimpering very early in life are often seemingly unintentional, but there are two reasons, we think, to keep them with the universals. First, beyond the first half year, crying and whimpering appear to acquire a much more obvious intentionality, with parents reporting across our many longitudinal studies that their infants routinely use these negative sounds to get attention and to manipulate parental action. The same is true of course of whining (protophones with negative affect)—they seem to become much more intentional and socially manipulative with age. Perhaps the most significant cry researcher in the past 4 decades is James Green, who in personal communication, reported that by six months, crying shifts from being primarily a response to pain or hunger to involving a new type of acoustic pattern that can be portrayed as angry and manipulative. A Spanish group [4] has provided evidence that crying in both anger and fear increases beyond 8 months (although it can occur earlier), implying differentiation of crying with age, where infant control of the import of their crying increases with age. Because our data for the present paper come from the second year of life, it would seem that if intentionality is the key issue, it may well be that, even though it was only a small amount, the crying and whimpering we observed should be viewed as primarily intentionally communicative.**

**The second point concerns the inherent communicativeness of crying and whimpering whether or not they are intentional. Even presumably unintentional crying and whimpering in infants is a naturally selected signal to caregivers of need on the part of infants, and consequently it can be reasoned that crying and whimpering are designed as initiators of care. Parents also talk with their infants often while they cry or whimper (as a part of the attempt to calm or soothe), yielding an impression of social interactivity, although with negative sounds the pattern of vocalization is generally more simultaneous than alternating vocalization of parents and infants [5]. Infant laughter can similarly be argued to be interactive on purely empirical grounds. Both laughter and smiling occur overwhelmingly in face to face social situations.**

**Also, although it is still not widely recognized, it is now clear based on longitudinal research with both laboratory and all-day recordings in infant homes that the great majority of protophones are endogenously produced with no sign of intended interactivity nor of elicitation by caregivers [6, 7].**

**For both vocalizations and gestures, we have come to the conclusion, after rethinking inspired by R1’s commentary, that all of the events that we coded as universal should be considered as socially interactive. In fact it was a criterion of the gesture coding that universal gestures had to be socially interactive, because if not, they would have been categorized as non-social.**

Measures Sessions are approximately 20 mins (not exactly 20 mins). This makes it hard to interpret individual differences across participants. How close to 20 minutes are the sessions? If within a few seconds, this is fine. However, if discrepancies are significant, behaviours should be standardized as rates per minute (or rate per 20 mins if preferred) instead of as a raw frequency. The analysis should then be re-run with these standardized measures.

**This is a very good point. The data patterns did not change, but all the data have been recalculated based on the actual duration of each session coded for each infant at each age, and these are the data reported in the revision. The average duration of sessions was actually just over 20.5 minutes, with a standard deviation of 1.4 minutes. The data in the repository include the raw number of events from each infant at each age in each of the three illocutionary categories along with the durations of each session and the computed rates/min.**

Reliability

Coding The first paragraph of Coder Agreement and Outcomes section is unnecessary – this is simply reporting reliability statistics from a previous study – not the current study – and it’s not clear why this is relevant. I suggest simply omitting this.

**Shortened substantially just leaving explanation about the similar training and lead up to the coding for the present paper as based on BR2021.**

The final paragraph concerning categorisation of vocalization and gesture into the three functional categories requires more quantitative reliability metrics. I agree (by eyeballing the plot) that this probably shows pretty decent agreement, however, given the way in which the data was coded, it should be possible to calculate kappa reliability (if I have understood the coding procedure correctly). I.e., for the instances in which both coders agreed there was a vocalisation or a gesture – did they agree on the function (i.e., did they both select an illocution code that would be categorized as the same function?). Overall though, it should be noted that reliability correlations currently reported are solid (and my impression is that the kappas will equally demonstrate this) – I have no doubt that this coding has been conducted meticulously, but there do need to be missing metrics included to confirm this.

**Kappa values have been added as requested, and indeed they appear to be adequate (.8 for gesture .65 for vocalization).**

**Analysis G**EE does seem an appropriate method for the data, and likewise Mann-Whitney tests are appropriate in my understanding. The GEE models obtained need to be included in the paper in full (if even simply in tables in an Appendix).

**We have provided the GEE tests of model effects (Wald Chi-Square values and df’s as well as significance levels) in the text. The GEE models are provided in an Appendix as requested.**

**Data visualisation** Given that the authors rely heavily on the visualisation of the data to do much of the work of inference (and correctly so I think!), the visualisations need work. I have a few suggestions: Distributions of data: Ideally the distribution of the datapoints themselves needs to be included (not just 95% confidence intervals and a mean). Numerous ways exist to do this, for example violin plots, half violin plots, and/or plotting the individual data points. E.g., in figures 3, 4 and 5, individual points (representing a participant) could be plotted in red at each time point for vocalisations and in blue for gestures, with summary statistics (mean/CIs) overlaid, perhaps in tandem with violin plots or similar.

**We have opted for box and whisker plots or the rates per min, which we hope will satisfy R1’s concern. Also in the data posting in the repository, all the individual data points for each participant are provided so that any reader will be able to construct data distributions on their own either for raw numbers of communicative events in each illocutionary category or for rates per min.**

Model predictions. Equally, if GEE was implemented using an R package, it may be possible to plot model predictions (perhaps overlaid) on the figures automatically – which would be very informative. Of course it is possible to do manually, but this does require expertise. This is becoming much more of a standard approach to visualising data (i.e., raw scores – as you have here, overlaid with model estimates). This would really support the authors inferences from the data and I would strongly encourage that they attempt this. This would also remove the need for caveats to be included in the figure captions about what the model shows compared to what the figure seems to show (i.e., the confidence intervals relating to the raw data not the SE/confidence intervals of the model predictions). Figures 2 and 5. For hypothesis 2 a pie chart is not informative – as the data comes from individual participants. Please plot the data so that the range of proportions of gestures/vocalisations combined with eye gaze across participants is clear (i.e., in the same way as figures relating to hypothesis 1 would be fine, y axis being proportion). As currently plotted, the discrepancy between the data and the model results (especially with regard to 20m timepoints) is hard to interpret (i.e., why there is a main effect of modality, not an interaction with age). Likewise for figure 2, though there are error bars, perhaps consider plotting the individual data points with summary statistics overlaid as suggested above (i.e., not as a bar chart), so the distribution of individual participant data can be observed.

**We have provided new box and whiskers plotting as well as the individual participant data, which will allow readers to analyze the data and/or display them in additional ways.**

Data availability Currently the data for the study is not available. I agree fully that videos cannot be shared. It is perfectly possible however to have the data worksheet mentioned in the availability statement already uploaded into the OSF for example (it can be a private submission), with an anonymized link provided only for the purpose of review. Minor comments Fig 3. - Typo on y axis label (“Frequancy”). Also figure 3 error bars do not look correct and it’s a screenshot containing “plot area” in a box over the middle. I have suggested more substantial changes to figures above. Typo – in the Results paragraph headed “Hypothesis 1a”, the text refers to Figure 5 – this should be figure 3? Please include in the limitations section that this is still a relatively small sample size (in terms of unique participants). Please include line numbers in the next draft to facilitate navigating the text and providing feedback. When discussing possibility of vocalization to serve function of communication very early in development (e.g., page 2) consider citing Papaeliou et al., 2002 (10.1044/1092-4388(2002/024)) and/or Esteve-Gibert & Prieto 2012 (<https://doi.org/10.1017/S0305000912000359> ) as these seem relevant too. Note, I could not see tables in my copy of the manuscript. Whether this is an editorial mistake or submission mistake, I’m unsure (I contacted the journal to ask, but didn’t get a response before submitting my review to the deadline). I look forward to reviewing the content of the tables in the next round of review.

**Line numbers have been added. We have posted the data in Mendeley for your scrutiny prior to acceptance of the paper, and will make the data public thereafter.**

Reviewer 2

**INTRODUCTION**

The literature review is relevant. The paper is clearly written with bibliographic references on par with latest published research. On page 14 the authors provide a clear description of the different types of gestures. I was glad to this this section because it provided a clear pathway to the information following the introduction.

I suggest that the authors provide the term typically developing (experimental group) earlier in the paper.

**Added in the introductory paragraph**

**METHODOLOGY**

I am not clear on how toddlers were examined to exclude developmental/language challenges.  Dis children have any episodes of otitis media? Where they excluded? I mention this because under such circumstances even children and toddlers with neurotypical development use more communicative gestures to compensate for reduced vocal output and compromised hearing sensitivity.

**A paragraph has been added to provide details on these issues.**

We have added descriptive information in the text under participants. Only two of the 12 participants had notable difficulties with otitis media, both of them involving ear tubes.

Is there a rater-to-rater reliability score?

**Kappa agreement levels have been added under coder agreement.**

**RESULTS**

No comments. This is a qualitative examination and data are clearly presented in a via descriptive non-param stats.

**DISCUSSION**

The authors describe the important performance and provide adequate explanation of the findings.  The writing is clear and the manuscript is well-organized.

The authors provide adequate description of findings which are combined with plausible interpretations.

The novelty of the study rests on the fact that the findings contribute and enrich the  database related to vocalizations versus gestures.

**Thanks for the encouragement.**

[Note: HTML markup is below. Please do not edit.]

Reviewer's Responses to Questions

Comments to the Author

1. Is the manuscript technically sound, and do the data support the conclusions?

The manuscript must describe a technically sound piece of scientific research with data that supports the conclusions. Experiments must have been conducted rigorously, with appropriate controls, replication, and sample sizes. The conclusions must be drawn appropriately based on the data presented.

Reviewer #1: Yes

Reviewer #2: Yes

2. Has the statistical analysis been performed appropriately and rigorously?

Reviewer #1: Yes

Reviewer #2: Yes

3. Have the authors made all data underlying the findings in their manuscript fully available?

The [PLOS Data policy](https://nam02.safelinks.protection.outlook.com/?url=http%3A%2F%2Fwww.plosone.org%2Fstatic%2Fpolicies.action%23sharing&data=05%7C01%7Ckoller%40memphis.edu%7C7bc0ead9f70a40a2fb4608dbcf3bf9c3%7Cae145aeacdb2446ab05a7858dde5ddba%7C0%7C0%7C638331629670906748%7CUnknown%7CTWFpbGZsb3d8eyJWIjoiMC4wLjAwMDAiLCJQIjoiV2luMzIiLCJBTiI6Ik1haWwiLCJXVCI6Mn0%3D%7C3000%7C%7C%7C&sdata=V9YAPT%2BFtn5MnTbFRBB6V7WETPkAA41j8uTBTzWXsfM%3D&reserved=0) requires authors to make all data underlying the findings described in their manuscript fully available without restriction, with rare exception (please refer to the Data Availability Statement in the manuscript PDF file). The data should be provided as part of the manuscript or its supporting information, or deposited to a public repository. For example, in addition to summary statistics, the data points behind means, medians and variance measures should be available. If there are restrictions on publicly sharing data—e.g. participant privacy or use of data from a third party—those must be specified.

Reviewer #1: No

Reviewer #2: Yes

4. Is the manuscript presented in an intelligible fashion and written in standard English?

PLOS ONE does not copyedit accepted manuscripts, so the language in submitted articles must be clear, correct, and unambiguous. Any typographical or grammatical errors should be corrected at revision, so please note any specific errors here.

Reviewer #1: Yes

Reviewer #2: Yes

5. Review Comments to the Author

Please use the space provided to explain your answers to the questions above. You may also include additional comments for the author, including concerns about dual publication, research ethics, or publication ethics. (Please upload your review as an attachment if it exceeds 20,000 characters)

Reviewer #1: Please see attached comments (in pdf form). Overall, my review is positive, but there are revisions that need to be made in order to get the manuscript into publishable form. I look forward to the next round of review.

Reviewer #2: I would like to thank you for giving me the opportunity to review this interesting and well-written piece of work. As per your request, here is the review of PONE-D-23-20708 entitled Frequencies and functions of vocalizations and gestures in the second year of life”. My recommendation is “Acceptance with minor changes”. My comments are mostly related to methodological and stylistic issues. The paper focuses on the critical theme related to the importance of gestures vis a vis vocalization as early indications of the origins of language. It follows a previously conducted study, with the current paper adding and supporting evidence of their theoretical EVO-DEVO framework. The theoretical framework of observation highlights the fact that more than ¾ of gestures across these second-year data were deictics (e.g., pointing and reaching), acts that while significant in supporting the establishment of referential vocabulary in both spoken and signed languages, are not signs, but have single universal deictic functions in the here and now. In contrast, words and signs are functionally flexible, making possible reference to abstractions that are not bound to any “particular illocutionary force nor to the here and now”. This topic is long overdue given the scarcity of data available from such topics related to the origins of language.

6. PLOS authors have the option to publish the peer review history of their article ([what does this mean?](https://nam02.safelinks.protection.outlook.com/?url=https%3A%2F%2Fjournals.plos.org%2Fplosone%2Fs%2Feditorial-and-peer-review-process%23loc-peer-review-history&data=05%7C01%7Ckoller%40memphis.edu%7C7bc0ead9f70a40a2fb4608dbcf3bf9c3%7Cae145aeacdb2446ab05a7858dde5ddba%7C0%7C0%7C638331629670906748%7CUnknown%7CTWFpbGZsb3d8eyJWIjoiMC4wLjAwMDAiLCJQIjoiV2luMzIiLCJBTiI6Ik1haWwiLCJXVCI6Mn0%3D%7C3000%7C%7C%7C&sdata=4bswNOtSOS7wcH6NMcP8ReVuv2QiGhgDzBxuXlwXeFo%3D&reserved=0)). If published, this will include your full peer review and any attached files.


If you choose “no”, your identity will remain anonymous but your review may still be made public.


Do you want your identity to be public for this peer review? For information about this choice, including consent withdrawal, please see our [Privacy Policy](https://nam02.safelinks.protection.outlook.com/?url=https%3A%2F%2Fwww.plos.org%2Fprivacy-policy&data=05%7C01%7Ckoller%40memphis.edu%7C7bc0ead9f70a40a2fb4608dbcf3bf9c3%7Cae145aeacdb2446ab05a7858dde5ddba%7C0%7C0%7C638331629670906748%7CUnknown%7CTWFpbGZsb3d8eyJWIjoiMC4wLjAwMDAiLCJQIjoiV2luMzIiLCJBTiI6Ik1haWwiLCJXVCI6Mn0%3D%7C3000%7C%7C%7C&sdata=7Fu1WczG4ew7uSE%2Fmm6CvL8S%2BJdQ3xypcFkS%2BRf9wJk%3D&reserved=0).

Reviewer #1: No

Reviewer #2: No

[NOTE: If reviewer comments were submitted as an attachment file, they will be attached to this email and accessible via the submission site. Please log into your account, locate the manuscript record, and check for the action link "View Attachments". If this link does not appear, there are no attachment files.]

While revising your submission, please upload your figure files to the Preflight Analysis and Conversion Engine (PACE) digital diagnostic tool, <https://pacev2.apexcovantage.com/>. PACE helps ensure that figures meet PLOS requirements. To use PACE, you must first register as a user. Registration is free. Then, login and navigate to the UPLOAD tab, where you will find detailed instructions on how to use the tool. If you encounter any issues or have any questions when using PACE, please email PLOS at [figures@plos.org](mailto:figures@plos.org). Please note that Supporting Information files do not need this step.

*In compliance with data protection regulations, you may request that we remove your personal registration details at any time.* [*(Remove my information/details)*](https://nam02.safelinks.protection.outlook.com/?url=https%3A%2F%2Fwww.editorialmanager.com%2Fpone%2Flogin.asp%3Fa%3Dr&data=05%7C01%7Ckoller%40memphis.edu%7C7bc0ead9f70a40a2fb4608dbcf3bf9c3%7Cae145aeacdb2446ab05a7858dde5ddba%7C0%7C0%7C638331629670906748%7CUnknown%7CTWFpbGZsb3d8eyJWIjoiMC4wLjAwMDAiLCJQIjoiV2luMzIiLCJBTiI6Ik1haWwiLCJXVCI6Mn0%3D%7C3000%7C%7C%7C&sdata=8imCF60NT4%2Ff2rTpzd7E2qZGzaFnyaYrx7j1mu%2FAXO4%3D&reserved=0)*. Please contact the publication office if you have any questions.*

1.	Sroufe L, Waters E. The ontogenesis of smiling and laughter: A perspective on the organization of development in infancy. Psych Rev. 1976;83:173-89.

2.	Sroufe L, Wunsch J. The development of laughter in the first year of life. Child Development. 1972;43:1326-44.

3.	Sroufe LA. Emotional Development:The Organization of Emotional Life in the Early Years. Cambridge: Cambridge University Press; 1995.

4.	Chóliz M, Fernández-Abascal EG, Martínez-Sánchez F. Infant crying: pattern of weeping, recognition of emotion and affective reactions in observers. The Spanish journal of psychology. 2012;15(3):978-88.

5.	Yoo H, Bowman DD, Oller DK. The origin of protoconversation: An examination of caregiver responses to cry and speech-like vocalizations. Frontiers in Psychology. 2018;24:1-15.

6.	Long HL, Bowman DD, Yoo H, Burkhardt-Reed MM, Bene ER, Oller DK. Social and endogenous infant vocalizations. PLOS ONE. 2020;15(8):e0224956.

7.	Oller DK, Ramsay G, Long HL, Griebel U. Protophones, the precursors to speech, dominate the human infant vocal landscape. Philosophical Transactions of the Royal Society B: Biological Sciences. 2021;376(1836):20200255.
