# Supplementary material for: Frequencies and functions of vocalizations and gestures in the second year of life
Source: PLoS One. 2025 Jan 9;20(1):e0308760. doi: 10.1371/journal.pone.0308760 (PMC11717318; doi:10.1371/journal.pone.0308760)
Supplement: Attachment — Submitted filename: PLoS One Re-review 21_03_2024.docx [file pone.0308760.s005.docx]

I’d like to thank the authors for their comprehensive and constructive response to the review. The authors have updated the literature review to present a more balanced view of the current opinion in the field, recalculated behaviours as rates (and subsequently re-run analyses – which I appreciate was a more substantial job), revised and added to the reliability information, included GEE model outputs, and they have deposited data in Mendeley. This is all great and I am very satisfied with this – thanks again to the authors.

I agree that given a low proportion of crying, whimpering and laughter that the argument over their inclusion with conversational protophones is perhaps somewhat moot, and they have made the case convincingly that these could even be considered (if not intentional) but socially interactive. I welcome their rethink that universal vocalisations (as gestures) should be considered as socially interactive, which I think is a very sensible and justified strategy. It wasn’t my initial intention – but I think it’s a useful revision, and well argued. So I am also satisfied on this point, and I’m really grateful that the authors took the time to set out their case.

Data visualization has been much improved. The box and whisker plots are very appropriate and really give a clear story of the data. My only outstanding concern with the plots is that often error bars (and even the boxes themselves) disappear below the x axis. I am not sure how this has come about. Obviously it should not be possible for rate per minute values (as in Figures 2 to 5) or proportions (as in Figure 6) to be less than 0.
