# Supplementary material for: Frequencies and functions of vocalizations and gestures in the second year of life
Source: PLoS One. 2025 Jan 9;20(1):e0308760. doi: 10.1371/journal.pone.0308760 (PMC11717318; doi:10.1371/journal.pone.0308760)
Supplement: Attachment — Submitted filename: Response to Reviewers.docx [file pone.0308760.s006.docx]

**Responses to Reviewers in Boldface**

PONE-D-23-20708R1

Frequencies and functions of vocalizations and gestures in the second year of life

PLOS ONE

Dear Dr. Burkhardt-Reed,

Thank you for submitting your manuscript to PLOS ONE. After careful consideration, we feel that it has merit but does not fully meet PLOS ONE’s publication criteria as it currently stands. Therefore, we invite you to submit a revised version of the manuscript that addresses the points raised during the review process.

Please submit your revised manuscript by Jul 12 2024 11:59PM. If you will need more time than this to complete your revisions, please reply to this message or contact the journal office at [plosone@plos.org](mailto:plosone@plos.org). When you're ready to submit your revision, log on to <https://www.editorialmanager.com/pone/> and select the 'Submissions Needing Revision' folder to locate your manuscript file.

Please include the following items when submitting your revised manuscript:

- A rebuttal letter that responds to each point raised by the academic editor and reviewer(s). You should upload this letter as a separate file labeled 'Response to Reviewers'.
- A marked-up copy of your manuscript that highlights changes made to the original version. You should upload this as a separate file labeled 'Revised Manuscript with Track Changes'.
- An unmarked version of your revised paper without tracked changes. You should upload this as a separate file labeled 'Manuscript'.

If you would like to make changes to your financial disclosure, please include your updated statement in your cover letter. Guidelines for resubmitting your figure files are available below the reviewer comments at the end of this letter.

If applicable, we recommend that you deposit your laboratory protocols in protocols.io to enhance the reproducibility of your results. Protocols.io assigns your protocol its own identifier (DOI) so that it can be cited independently in the future. For instructions see: [https://journals.plos.org/plosone/s/submission-guidelines#loc-laboratory-protocols](https://nam02.safelinks.protection.outlook.com/?url=https%3A%2F%2Fjournals.plos.org%2Fplosone%2Fs%2Fsubmission-guidelines%23loc-laboratory-protocols&data=05%7C02%7Ckoller%40memphis.edu%7Caf62afaa96054468ca5708dc7f332692%7Cae145aeacdb2446ab05a7858dde5ddba%7C0%7C0%7C638525105413661325%7CUnknown%7CTWFpbGZsb3d8eyJWIjoiMC4wLjAwMDAiLCJQIjoiV2luMzIiLCJBTiI6Ik1haWwiLCJXVCI6Mn0%3D%7C0%7C%7C%7C&sdata=l08PDXm3LVkdQzznRz6ZWZ3%2FEp733DtrAvaDF4tehP8%3D&reserved=0). Additionally, PLOS ONE offers an option for publishing peer-reviewed Lab Protocol articles, which describe protocols hosted on protocols.io. Read more information on sharing protocols at [https://plos.org/protocols?utm_medium=editorial-email&utm_source=authorletters&utm_campaign=protocols](https://nam02.safelinks.protection.outlook.com/?url=https%3A%2F%2Fplos.org%2Fprotocols%3Futm_medium%3Deditorial-email%26utm_source%3Dauthorletters%26utm_campaign%3Dprotocols&data=05%7C02%7Ckoller%40memphis.edu%7Caf62afaa96054468ca5708dc7f332692%7Cae145aeacdb2446ab05a7858dde5ddba%7C0%7C0%7C638525105413671913%7CUnknown%7CTWFpbGZsb3d8eyJWIjoiMC4wLjAwMDAiLCJQIjoiV2luMzIiLCJBTiI6Ik1haWwiLCJXVCI6Mn0%3D%7C0%7C%7C%7C&sdata=4Rh9CQDGsfvLARdX70OuFuZZf%2FCvCNz6JE6KwyBXALo%3D&reserved=0).

We look forward to receiving your revised manuscript.

Kind regards,

Elena Theodorou
Academic Editor
PLOS ONE

Journal Requirements:

Please review your reference list to ensure that it is complete and correct. If you have cited papers that have been retracted, please include the rationale for doing so in the manuscript text, or remove these references and replace them with relevant current references. Any changes to the reference list should be mentioned in the rebuttal letter that accompanies your revised manuscript. If you need to cite a retracted article, indicate the article’s retracted status in the References list and also include a citation and full reference for the retraction notice.


[Note: HTML markup is below. Please do not edit.]

Reviewers' comments:

Reviewer's Responses to Questions

**FROM R1**

I’d like to thank the authors for their comprehensive and constructive response to the review. The authors have updated the literature review to present a more balanced view of the current opinion in the field, recalculated behaviours as rates (and subsequently re-run analyses – which I appreciate was a more substantial job), revised and added to the reliability information, included GEE model outputs, and they have deposited data in Mendeley. This is all great and I am very satisfied with this – thanks again to the authors.

I agree that given a low proportion of crying, whimpering and laughter that the argument over their inclusion with conversational protophones is perhaps somewhat moot, and they have made the case convincingly that these could even be considered (if not intentional) but socially interactive. I welcome their rethink that universal vocalisations (as gestures) should be considered as socially interactive, which I think is a very sensible and justified strategy. It wasn’t my initial intention – but I think it’s a useful revision, and well argued. So I am also satisfied on this point, and I’m really grateful that the authors took the time to set out their case.

Data visualization has been much improved. The box and whisker plots are very appropriate and really give a clear story of the data. My only outstanding concern with the plots is that often error bars (and even the boxes themselves) disappear below the x axis. I am not sure how this has come about. Obviously it should not be possible for rate per minute values (as in Figures 2 to 5) or proportions (as in Figure 6) to be less than 0.

**Comments to the Author**

1. If the authors have adequately addressed your comments raised in a previous round of review and you feel that this manuscript is now acceptable for publication, you may indicate that here to bypass the “Comments to the Author” section, enter your conflict of interest statement in the “Confidential to Editor” section, and submit your "Accept" recommendation.

Reviewer #1: (No Response)

Reviewer #3: (No Response)

2. Is the manuscript technically sound, and do the data support the conclusions?

The manuscript must describe a technically sound piece of scientific research with data that supports the conclusions. Experiments must have been conducted rigorously, with appropriate controls, replication, and sample sizes. The conclusions must be drawn appropriately based on the data presented.

Reviewer #1: Yes

Reviewer #3: Partly

3. Has the statistical analysis been performed appropriately and rigorously?

Reviewer #1: Yes

Reviewer #3: Yes

4. Have the authors made all data underlying the findings in their manuscript fully available?

The [PLOS Data policy](https://nam02.safelinks.protection.outlook.com/?url=http%3A%2F%2Fwww.plosone.org%2Fstatic%2Fpolicies.action%23sharing&data=05%7C02%7Ckoller%40memphis.edu%7Caf62afaa96054468ca5708dc7f332692%7Cae145aeacdb2446ab05a7858dde5ddba%7C0%7C0%7C638525105413678605%7CUnknown%7CTWFpbGZsb3d8eyJWIjoiMC4wLjAwMDAiLCJQIjoiV2luMzIiLCJBTiI6Ik1haWwiLCJXVCI6Mn0%3D%7C0%7C%7C%7C&sdata=mN0FD9r01tEGEtIWSeijcEdtKeiXPPR6NU2%2B3CaTUyU%3D&reserved=0) requires authors to make all data underlying the findings described in their manuscript fully available without restriction, with rare exception (please refer to the Data Availability Statement in the manuscript PDF file). The data should be provided as part of the manuscript or its supporting information, or deposited to a public repository. For example, in addition to summary statistics, the data points behind means, medians and variance measures should be available. If there are restrictions on publicly sharing data—e.g. participant privacy or use of data from a third party—those must be specified.

Reviewer #1: Yes

Reviewer #3: Yes

5. Is the manuscript presented in an intelligible fashion and written in standard English?

PLOS ONE does not copyedit accepted manuscripts, so the language in submitted articles must be clear, correct, and unambiguous. Any typographical or grammatical errors should be corrected at revision, so please note any specific errors here.

Reviewer #1: Yes

Reviewer #3: Yes

6. Review Comments to the Author

Please use the space provided to explain your answers to the questions above. You may also include additional comments for the author, including concerns about dual publication, research ethics, or publication ethics. (Please upload your review as an attachment if it exceeds 20,000 characters)

**Author responses to the reviewer comments are in boldface below.**

Reviewer #1: Please see my attached comments for one final minor revision. Overall, again my review is very positive.

**Thanks for your comments and noticing the oddity in the figures. They have been corrected. We have provided an explanation for why the minimum whiskers cannot be displayed when the minimum is at zero.**

Reviewer #3: The paper in general is very well written. I do have a few considerations though, regarding methodology and the conclusions. My comments are shown below:

• Line 96: it has been reasoned that in the massive amounts of vocal explorations …

**Thanks for noticing the ungrammaticality of the sentence. We found and corrected it. The sentence now reads: “It has been reasoned that the massive amount of vocal exploration in human infants forms a foundation for language.”**

METHODOLOGY/RESULTS

• I have an issue with the inclusion of the category “social plauful” in Universal vocalizations. Although animal sounds or object sounds are echopoietic, they are still conventional across languages, so they are the product of learning/associations. I would have liked to know what the percentage of this category is in Universal Vocalizations across ages, and also I suspect that the fact that there is no significant difference between gestures and vocalizations in the Universal category is due to the fact that this subcategory was wrongly included in Universal instead of conventional. When this happens the difference should become evident.

**The reviewer deserves a big thank you for this comment. Table 2 had an error that misled the reviewer and would surely have misled other readers. In fact the “social playful” category for vocalization was eliminated from the Universal domain prior to our final sorting of data into categories, but we forgot to eliminate the mistaken line in Table 2. There were very few instances of animal or object sounds, but in the final analysis they were all assigned to the Conventional vocal group, because we noticed, like the reviewer, that they were at least somewhat language-specific and thus should be thought of as learned. Table 2 has been revised accordingly, eliminating the offending row. Since the data were actually sorted correctly, the relation between the amount of Universal vocalization and Universal gesture was reported correctly, in accord with the reviewer’s opinion on how the sorting should have been done.**

• The fact that the two coders agree only at .8 and .65 regarding social/non-social interactions creates a problem. The percentage is very low. (line 523/524). This suggests highly ambiguous data and problematic categories. Can you please explain how you handled this problem?

**The reviewer may know that kappa does not actually represent a percentage, but a special measure of agreement. The standard interpretation is that values between 0.01–0.20 represent slight agreement, 0.21–0.40 are seen as fair, 0.41– 0.60 as moderate, 0.61–0.80 as substantial, and 0.81–1.00 as almost perfect agreement. Both the kappa values are thus in the “substantial” range. Percent agreement is nowadays largely disregarded as a measure of agreement because it can be misleading if expected values in cells of a comparison are widely different, as in the present case—kappa corrects for such imbalances.**

**In our opinion, the most important agreement issue for our paper is the similarity of the distribution of identified categories across the two coders for the three domains (Non-social, Universal, Conventional) as portrayed in Figure 1. For gesture there were 170 items categorized with only 4 that were not agreed upon. For vocalization there were many more disagreements (73/365), but the outcome still corresponds to 80% agreement (which sounds better than .65 kappa, but that’s because kappa corrects for the imbalance of expected values). A 2x3 chi-square produced a wildly significant agreement (p < .0001) between the coders on vocalization categorization into the three domains. Whenever agreement is above chance, it is justified to conduct comparative analyses across groups of data, because if there are significant differences between the groups, there is good reason to trust them, since coder disagreement constitutes noise in the data and would produce Type II, not Type I error. In essence the more noise there is in the data, the harder it is to detect real group differences.**

**Finally, we hope the reviewer will notice our explanation in the main text about the primary source of the differences between coders regarding categorization of vocalizations into the domains. There is inherent difficulty in getting high agreement between coders on whether an infant vocalization is directed to an eliciting adult (whether it is non-social or social-interactive-universal) in a circumstance where the infant attention is varying moment to moment. This is not a fault of our method, but an inherent characteristic of the complexity in the actions of the infant and the parent during interaction, which involves eye contact, timing and other contextual factors, all of which are not only influencing each other but also varying moment to moment. One cannot and should not expect very high agreement among coders on all factors of judgments about infant intentions in such a circumstance. Still our agreement level justifies our analyses.**

• I cannot see any figures in the Revision 1 attachment. Seeing them in the initial submission, please add descriptions of the axes in all figures

**We do not understand why it has been so difficult for all the relevant files to be made available to the reviewers. You are not the first to mention the problem. We will do whatever we can to ensure you have all the files necessary.**

DISCUSSION
• I also have an issue with the discussion about what constitutes a gesture and a general utilitarian movement. Also I would like to bring into this discussion the issue of development of fine motor skills which takes time for infants, even in the first year of life.

In other words, gestures involve fine motor movements, which take longer to develop than gross motor movements and sounds (a bit like having protophones and language sounds). So although there are movements with the hands, they are not considered gestures, as the latter take long to be produced in a controlled manner.

**This suggestion has arisen in prior comments on the previous paper, as well as in presentations on our work. We have added a paragraph in the Discussion section explaining our opinion. The new paragraph reads as follows:**

**“It might be suggested that the higher rate of vocalization than gesture in early life can be accounted for by the fact that fine motor control of the arms, hands and fingers does not develop until later than phonatory control. But the suggestion in fact merely restates the finding rather than explaining it. Yes, the motor control of protophones occurs before the motor control of arms, but the appropriate question is why? We contend that an evolutionary explanation is needed and that the fitness signaling theory provides the necessary reasoning. In accord with the theory, it was and is because of the altriciality of infants in the human line (their slow and long-term development of head control, arm control, locomotion, and fine control of the extremities) that there has been selection pressure on them to signal their wellness effectively enough to elicit the long-term care they require because of altriciality. Phonation, according to the theory, long ago in hominin history became the target of that selection pressure, and in that way phonatory activity in humans became a precocial feature of hominins and thus laid a foundation for later developments leading to language. At a later point in evolution, complex gesture and ultimately sign language can be seen as having become, in accord with the reasoning, important secondary developments to the primary evolution of language in the vocal domain.”**

**The following additional reasoning might be useful as a response to the reviewer’s suggestion.**

**It is still not widely realized that infants are very active independently in production of phonation. As cited in the ms, even preterm infants not yet released from intensive care, produce highly differentiated phonatory patterns (Oller et al., 2019). In a sense, our earlier publication (Burkhardt-Reed et al., 2021) and those of others showing motor control of phonation proceeds faster than finger and hand motor control (that would be manifest if gesture were more prominent in early development) are the evidence that justifies the reviewer’s observation: phonatory development comes earlier than the fine motor control of gesture. The real issue is why fine motor control of phonation should develop faster than fine motor control of the hands and limbs. Notice that control of the supraglottal articulators comes later in development than phonatory control—canonical babbling, for example, begins in the second half year** **(Lang et al., 2019; Lohmander et al., 2020; Stoel-Gammon, 1992)—and it is not clear how to scale the rate of development of supraglottal articulation in comparison with that of fine motor control of the hands and fingers. What is eminently clear is that phonation control in humans comes earlier than either fine supraglottal articulatory control or fine motor control of the hands.**

**One might say that humans (in comparison with many other mammals and especially other primates) are very altricial with regard to such matters as fine finger and hand control, locomotion, head control, and posture. But they are very precocial with regard to phonation. In fact their control of phonation is not only precocial, it is largely unique among primates at any age—other primates have been found to have little if any voluntary control of phonation (Hauser, 1996). Training of any non-human primate to produce any phonatory vocalization on command has proven extremely difficult and success at any level may require years of operant conditioning** **(Hopkins et al., 2011). In contrast, training of supraglottal acts such as blowing raspberries or sticking the tongue out is easy. An evolutionary interpretation of these facts might be simply that the human line has been naturally selected to have not only the ability but the inclination to produce phonation and to explore it from birth. The fitness signaling theory offers an explanation as to how the human phonatory capability could have been naturally selected (Griebel & Oller, 2024; Locke, 2006). One might say that the overall altriciality of the human infant, requiring very long term provisioning and protection,was compensated for through natural selection of a way that infants could elicit long term care by vocal fitness signaling in the form of exploratory phonation.**

• A final issue concerns a point acknowledged in the limitations section – there are no data from deaf children and this suggests that we cannot know whether the patterns observed are due to an evolutionary reason or whether a special emphasis on vocalizations is the result of communicative reinforcement from parent interactions throughout the first year. So if we are going to talk about an evolutionary perspective emerging from the current data, we need to account for the fact that parents respond to vocalizations from day 1 but not to gestures. So how do we know that the current data, and preference for vocalizations over gestures, are the result of a genetic/evolutionary basis of language and not just conditioning/reinforcement for sounds instead of gestures?

**Indeed, we would love to make a report on vocal and gestural development in congenitally deaf infants. But the relevant data are not available to us.**

**On the conditioning suggestion, even if conditioning played a big role in early rates of gesture and vocalization (which seems doubtful, see next paragraphs), vocalization could be reinforced vastly more often than gesture simply because vocalization occurs shortly after birth at high rates while gesture is essentially absent shortly after birth. For gesture, there is virtually nothing to reinforce, while for protophones, there tend to be 4-5 per minute every waking hour (Oller et al., 2021).**

**But widely available data (especially since all-day recordings have been available) contradict the widespread belief that human infants vocalize largely in response to caregiver elicitation and that they learn vocalization (perhaps even phonation) through operant conditioning/parent reinforcement (Long et al., 2020; Long et al., 2019). We now know that the vast majority (more than 90%) of infant protophones are produced in the absence of any elicitation, and that they are very rarely directed toward caregivers in the natural environment of the home. There is no solid evidence that prior to the end of the first year, infants ever produce sounds on the basis of adaptation to caregiver vocalizations that are not themselves imitations by the caregivers of sounds the caregivers realize are already in the infant repertoires. The widespread belief in vocal imitation as the source of infant protophones is speculation and has no basis in fact that we know of. Experimental studies that claim to show infant vocal imitation prior to the end of the first year have been, we think, widely overinterpreted. In general what they show is that infants at various points across the first year can be experimentally induced to produce sounds already in their repertoires given vocal stimulation by sounds similar to those in their repertoires (e.g., Goldstein & Schwade, 2008; Kessen et al., 1979; Kugiumutzakis, 1999), but we know of no evidence of young infants being stimulated to produce sounds not in their endogenously developed repertoires.**

**So where do the infant protophones come from? They appear to be the products of endogenous exploration, overwhelmingly. When they do occur in face-to-face interaction, they show signs of adaptation to make them especially good signals of fitness (Long et al., 2022), but they do not show signs of imitation until the end of the first year, and even then only rarely. Vocabulary learning of course depends on vocal imitation, but that is a phenomenon that is extremely limited until the second year.**

**So, although the evidence is largely observational rather than experimental, it supports the idea that vocal development is overwhelmingly endogenous in the first year.**

**Of course there is no way to fully rule out the possibility that there is a role for differential reinforcement in the dramatic difference between rates of vocalization and gesture in early life.**

**If the editor thinks there should be a statement acknowledging a role for differential reinforcement cannot be completely ruled out, we will add it.**

7. PLOS authors have the option to publish the peer review history of their article ([what does this mean?](https://nam02.safelinks.protection.outlook.com/?url=https%3A%2F%2Fjournals.plos.org%2Fplosone%2Fs%2Feditorial-and-peer-review-process%23loc-peer-review-history&data=05%7C02%7Ckoller%40memphis.edu%7Caf62afaa96054468ca5708dc7f332692%7Cae145aeacdb2446ab05a7858dde5ddba%7C0%7C0%7C638525105413685218%7CUnknown%7CTWFpbGZsb3d8eyJWIjoiMC4wLjAwMDAiLCJQIjoiV2luMzIiLCJBTiI6Ik1haWwiLCJXVCI6Mn0%3D%7C0%7C%7C%7C&sdata=YAcAnhbzPV7d7JLnTxIyq0vFbMYHOf4MUzympNYDh6U%3D&reserved=0)). If published, this will include your full peer review and any attached files.


If you choose “no”, your identity will remain anonymous but your review may still be made public.


**Do you want your identity to be public for this peer review?** For information about this choice, including consent withdrawal, please see our [Privacy Policy](https://nam02.safelinks.protection.outlook.com/?url=https%3A%2F%2Fwww.plos.org%2Fprivacy-policy&data=05%7C02%7Ckoller%40memphis.edu%7Caf62afaa96054468ca5708dc7f332692%7Cae145aeacdb2446ab05a7858dde5ddba%7C0%7C0%7C638525105413691974%7CUnknown%7CTWFpbGZsb3d8eyJWIjoiMC4wLjAwMDAiLCJQIjoiV2luMzIiLCJBTiI6Ik1haWwiLCJXVCI6Mn0%3D%7C0%7C%7C%7C&sdata=yGkyH%2Bfb2BgovAYn%2FWapmj%2FBRoTLv6v90VxRBsWLCUY%3D&reserved=0).

Reviewer #1: No

Reviewer #3: **Yes:** Loukia Taxitari

Burkhardt-Reed, M. M., Long, H. L., Bowman, D. D., Bene, E. R., & Oller, D. K. (2021). The origin of language and relative roles of voice and gesture in early communication development. *Infant Behav Dev*, *65*, 101648. <https://doi.org/10.1016/j.infbeh.2021.101648>

Goldstein, M. H., & Schwade, J. A. (2008). Social feedback to infants’ babbling facilitates rapid phonological learning. *Psychological Science*, *19*, 515-522.

Griebel, U., & Oller, D. K. (2024). From emotional signals to symbols. *Frontiers in Pychology*. <https://doi.org/doi:> 10.3389/fpsyg.2024.1135288

Hauser, M. (1996). *The evolution of communication*. MIT.

Hopkins, W. D., Taglialatela, J., & Leavens, D. A. (2011). Do apes have voluntary control of their vocalizations and facial expressions? In A. Vilain, J. L. Schwartz, C. Abry, & J. Vauclair (Eds.), *Primate communication and human language* (pp. 206-226). John Benjamins Publishing.

Kessen, W., Levine, J., & Wendrich, K. (1979). The imitation of pitch in infants. *Infant Behavior and Development*, *2*, 93-99.

Kugiumutzakis, G. (1999). Genesis and development of early infant mimesis to facial and vocal models. In J. Nadel & G. Butterworth (Eds.), *Imitation in infancy. Cambridge studies in cognitive perceptual development* (pp. 36-59). Cambridge University Press.

Lang, S., Bartl-Pokorny, K. D., Pokorny, F. B., Garrido, D., Mani, N., Fox-Boyer, A. V., . . . Marschik, P. B. (2019). Canonical Babbling: A Marker for Earlier Identification of Late Detected Developmental Disorders? *Current Developmental Disorders Reports*, *6*(3), 111-118. <https://doi.org/10.1007/s40474-019-00166-w>

Locke, J. L. (2006). Parental selection of vocal behavior: Crying, cooing, babbling, and the evolution of language. *Human Nature*, *17*, 155-168. <https://doi.org/https://link.springer.com/article/10.1007/s12110-006-1015-x>

Lohmander, A., Westberg, L. R., Olsson, S., Tengroth, B. I., & Flynn, T. (2020). Canonical Babbling and Early Consonant Development Related to Hearing in Children With Otitis Media With Effusion With or Without Cleft Palate. *Cleft Palate Craniofac J*, 1055665620966198. <https://doi.org/10.1177/1055665620966198>

Long, H. L., Bowman, D. D., Yoo, H., Burkhardt-Reed, M. M., Bene, E. R., & Oller, D. K. (2020). Social and endogenous infant vocalizations. *PLoS One*, *15*(8), e0224956. <https://doi.org/10.1371/journal.pone.0224956>

Long, H. L., Oller, D. K., & Bowman, D. D. (2019). Reliability of Listener Judgments of Infant Vocal Imitation [Original Research]. *Frontiers in Psychology*, *10*(1340). <https://doi.org/10.3389/fpsyg.2019.01340>

Long, H. L., Ramsay, G., Bene, E. R., Burkhardt-Reed, M. M., & Oller, D. K. (2022). Perspectives on the origin of language: Infants vocalize most during independent vocal play but produce their most speech-like vocalizations during turn taking. *PLoS One*. <https://doi.org/https://doi.org/10.1371/journal.pone.0279395>

Oller, D. K., Caskey, M., Yoo, H., Bene, E. R., Jhang, Y., Lee, C.-C., . . . Vohr, B. (2019). Preterm and full term infant vocalization and the origin of language. *Scientific Reports*, *9*. <https://doi.org/https://doi.org/10.1038/s41598-019-51352-0>

Oller, D. K., Ramsay, G., Long, H. L., & Griebel, U. (2021). Protophones, the precursors to speech, dominate the human infant vocal landscape. *Philosophical Transactions of the Royal Society B: Biological Sciences*, *376*(1836), 20200255. <https://doi.org/doi:10.1098/rstb.2020.0255>

Stoel-Gammon, C. (1992). Prelinguistic vocal development: Measurement and prediction. In C. Ferguson, L. Menn, & C. Stoel-Gammon (Eds.), *Phonological development: Models, Research, Implications* (pp. 439-456). York Press, Inc.
